# Paracetamol therapy and outcome of critically ill patients: a multicenter retrospective observational study

**DOI:** 10.1186/s13054-015-0865-1

**Published:** 2015-04-13

**Authors:** Satoshi Suzuki, Glenn M Eastwood, Michael Bailey, David Gattas, Peter Kruger, Manoj Saxena, John D Santamaria, Rinaldo Bellomo

**Affiliations:** Austin Hospital, 145 Studley Rd, Heidelberg, Victoria 3084 Australia; Okayama University Hospital, 700-0082 Okayama Prefecture, Okayama 1-1-1, Japan; Australian and New Zealand Intensive Care Research Centre, Alfred Centre, 53 Commercial Rd, Melbourne, Victoria 3004 Australia; Royal Prince Alfred Hospital, Missenden Rd, Camperdown, NSW 2050 Australia; Princess Alexandra Hospital, 237 Ipswich Rd, Wooloongabba, QLD 4102 Australia; St George Hospital, Gray St, Kogarah, NSW 2217 Australia; St Vincent’s Hospital, 59 Victoria Parade, Fitzroy, Victoria 3065 Australia

## Abstract

**Introduction:**

In this study, we aimed to examine the association between paracetamol administration in the intensive care unit (ICU) and mortality in critically ill patients.

**Methods:**

We conducted a multicenter retrospective observational study in four ICUs. We obtained information on paracetamol use, body temperature, demographic, clinical and outcome data from each hospital’s clinical information system and admissions and discharges database. We performed statistical analysis to assess the association between paracetamol administration and hospital mortality.

**Results:**

We studied 15,818 patients with 691,348 temperature measurements at 4 ICUs. Of these patients, 10,046 (64%) received at least 1 g of paracetamol. Patients who received paracetamol had lower in-hospital mortality (10% vs. 20%, *P* <0.001), and survivors were more likely to have received paracetamol (66% vs. 46%; *P* <0.001). However, patients treated with paracetamol were also more likely to be admitted to the ICU after surgery (70% vs. 51%; *P* <0.001) and/or after elective surgery (55% vs. 37%; *P* <0.001). In multivariate logistic regression analysis including a propensity score for paracetamol treatment, we found a significant and independent association between the use of paracetamol and reduced in-hospital mortality (adjusted odds ratio =0.60 (95% confidence interval (CI), 0.53 to 0.68), *P* <0.001). Cox proportional hazards analysis showed that patients who received paracetamol also had a significantly longer time to death (adjusted hazard ratio =0.51 (95% CI, 0.46 to 0.56), *P* <0.001). The association between paracetamol and decreased mortality and/or time to death was broadly consistent across surgical and medical patients. It remained present after adjusting for paracetamol administration as a time-dependent variable. However, when such time-dependent analysis was performed, the association of paracetamol with outcome lost statistical significance in the presence of fever and suspected infection and in patients in the lower tertiles of Acute Physiology and Chronic Health Evaluation II scores.

**Conclusions:**

Paracetamol administration is common in the ICU and appears to be independently associated with reduced in-hospital mortality and time to death after adjustment for multiple potential confounders and propensity score. This association, however, was modified by the presence of fever, suspected infection and lesser illness severity and may represent the effect of indication bias.

**Electronic supplementary material:**

The online version of this article (doi:10.1186/s13054-015-0865-1) contains supplementary material, which is available to authorized users.

## Introduction

Paracetamol, the acetaminophen prodrug, is a widely used analgesic and antipyretic drug in the intensive care unit (ICU) [1]. However, no randomized controlled trials have been performed to assess its use as an antipyretic in this setting in developed countries, and only limited studies of its effects or associations with outcome in critically ill patients overall have been conducted [2,3]. This lack of data is of potential concern because paracetamol may carry some risks. For example, it can act as a liver toxin by depleting its intracellular glutathione concentration through the effects of one of its metabolites, called *N*-acetyl-*p*-benzoquinone imine [4]. This effect is exacerbated by fasting, a condition commonly seen in ICU patients. Moreover, the toxic dose of paracetamol is highly variable. For example, after administration of a standard dose of 4 g/day for 2 to 3 weeks, an increase in alanine aminotransferase to about three times the normal value is common [5]. Thus, it is conceivable that when used in critically ill patients who have other risk factors for liver injury and/or are in a fasting state, paracetamol may lead to subclinical adverse effects that contribute to morbidity and mortality.

In addition, paracetamol is also likely to affect the febrile response to critical illness. Fever is common in critically ill patients [6,7] and may be deleterious, especially in ICU patients, by increasing metabolic rate, heart rate, cardiac work and catecholamine production [8-10]. Thus, by lowering metabolic demand, the antipyretic effect of paracetamol may have a protective effect. However, in the presence of infection, fever may be protective [11,12]. Thus, the potential balance between the beneficial and detrimental effects of paracetamol on body temperature may depend on the cause of fever [13-15]. Moreover, there remains substantial clinical uncertainty about the effect of pharmacologic antipyretic therapy on outcomes in critically ill patients in general [15].

Thus, paracetamol prescription is common [6,14] and may have important consequences. Despite such potential risks and/or benefits, little is known about the epidemiology and safety of paracetamol administration in the ICU. Observational studies are therefore desirable as a first step toward improved understanding and knowledge of paracetamol administration. Accordingly, we aimed to investigate the epidemiology and associations of paracetamol in a cohort of critically ill patients. In particular, we hypothesized that paracetamol administration might be common, associated with no overall differences in mortality in critically ill patients, but associated with increased risk of death in febrile patients with infection as the admission diagnosis.

## Materials and methods

### Study design

This study was a multicenter retrospective observational study conducted at the ICUs of Princess Alexandra Hospital (Brisbane), Royal Prince Alfred Hospital (Sydney), St George Hospital (Sydney) and St Vincent’s Hospital (Melbourne). These ICUs are all tertiary units in major metropolitan centers in Australia.

### Ethical approval

The data collection and data analysis were approved by the Austin Hospital Ethics Committee (approval H2010/04086) and by ethics committees in each of the participating hospitals (see Acknowledgements for details). As this study was deemed to be low risk, the need for informed consent was waived.

### Patients

We studied all adult patients admitted to the study ICUs during the period when electronic data capture of paracetamol and body temperature prescription was possible (from 6 January 2000 to 1 September 2010 in hospital A, from 1 January 2009 to 31 December 2010 in hospital B, from 1 July 2009 to 9 June 2011 in hospital C and from 1 July 2009 to 31 December 2012 in hospital D). We excluded readmission episodes and patients for whom data were not available for temperature, admission diagnosis or vital status at hospital discharge and patients with insufficient data for illness severity assessment.

We additionally extracted the following variables from the relevant unit databases: demographics, physiological parameters over the first 24 hours in the ICU, admission source, Acute Physiology and Chronic Health Evaluation (APACHE) II score, Simplified Acute Physiology Score II, body temperature during ICU stay (recorded by any route) and vital status at ICU and hospital discharge.

### Body temperature measurements

For all patients, all ICU temperature values (total of 691,348) during the entire ICU admissions were available. Measurement methods included tympanic, axillary, per rectum, esophageal and bladder temperature probes. Fever was defined as a body temperature >38.0°C [16-18].

### Statistical analyses

All analysis was performed using SAS version 9.4 software (SAS Institute, Cary, NC, USA) and STATA version 11.0 software (StataCorp, College Station, TX, USA). Continuous data are presented as mean (standard deviation) or medians (interquartile range) depending on the underlying distribution of the data. Categorical data are reported as proportions (n).

The primary outcome measure for this study was in-hospital mortality, and the secondary outcome was ICU mortality. Patients exposed to paracetamol (at least one dose either intravenously or via nasogastric tube or per rectum or orally) were first compared with those not exposed to paracetamol by means of univariate analysis, and time to event was assessed by log-rank test. The results are presented as Kaplan-Meier survival plots.

Multivariate analysis for in-hospital mortality using logistic regression and Cox proportional hazards regression were then conducted, and the results are expressed as odds ratios (ORs) or hazard ratios (HRs), respectively. Proportionality assumptions were determined both visually and by fitting interactions with the log of survival time.

Given the retrospective nature of the study design, to account for imbalance between patients who received paracetamol compared with those who did not, each patient’s propensity to receive paracetamol was included as a covariate in multivariate analysis. The propensity to receive paracetamol for each patient was determined by using a multivariable logistic regression with “paracetamol administration” or “no paracetamol administration” as the outcome. This model was constructed using both stepwise and backward selection procedures (with an inclusion *P*-value of 0.05) before a final assessment for clinical and biological plausibility. Variables included in the final propensity score model are displayed in Additional file 1: Table E1. This propensity score was used as a covariate in the multivariate models in conjunction with APACHE II score, hospital, surgical patient, infection as admission diagnosis, presence of fever, APACHE III admission diagnostic group as modified by the Australian and New Zealand Intensive Care Society Adult Patient Database [16], treatment limitation and paracetamol administration. Additional propensity score matching analysis was also performed by matching “paracetamol administration” with “no paracetamol administration,” with each patient matched to within ±5% of the propensity score.

In addition, to account for the competing risk of patients’ dying before having the opportunity to receive paracetamol, time to death was analyzed using Cox proportional hazards regression with paracetamol exposure (yes vs. no) treated as a time-dependent variable. However, as fever was not found to be time-dependent, it was not treated as such in the analysis.

For sensitivity analysis, the same analyses were performed in the following subgroups:

1. Surgical (post-operative) patients
2. Medical (non-operative) patients
3. Patients with and without fever (any temperature above and below 38°C, respectively)
4. Patients with and without fever (any temperature above and below 38.3C, respectively)
5. Patients with and without fever (any temperature above and below 38.5°C, respectively)
6. Patients with and without fever (any temperature above and below 39°C, respectively)
7. Patients with hypothermia (any temperature <35°C)
8. Medical patients with fever (any temperature >38°C) who had infection as admission diagnosis
9. Patients with and without cirrhosis
10. Patients who received paracetamol enterally
11. Patients who received paracetamol intravenously
12. Patients stratified by tertiles of APACHE II scores

Finally, to further decrease the impact of early deaths as well as early discharges, and as an additional sensitivity analysis, we used a cutoff time point of 20 hours when approximately two-thirds of patients had received exposure to paracetamol and close to 40% of patients had either died or been discharged. These patients were excluded from analysis. This cutoff point was selected without knowledge of patient outcomes and was based on the frequency distribution of paracetamol exposure.

In this cohort, for the purpose of sensitivity analysis, we retested the Cox proportional hazards model with paracetamol treatment as a time-dependent variable adjusting for hospital, diagnosis, APACHE III score, presence of treatment limitations orders, surgical admissions, infection-related admission and propensity to receive paracetamol. A two-sided *P*-value <0.01 was considered statistically significant.

## Results

### Patient characteristics

We screened 17,110 patients. We excluded 1,292 patients (missing diagnosis in 71; missing APACHE III score in 203, missing temperature in 19 and missing outcome in 972) and thus studied a total of 15,818 patients. Of these 10,046 patients (63.5%) were given at least 1 g of paracetamol with more than 90% of patients having their first exposure to paracetamol within the first 48 hours of ICU admission (Additional file 1: Figure E1).

The baseline characteristics, physiological data and outcomes for patients who did or did not receive paracetamol are shown in Table 1. As expected, there were significant imbalances between groups. In particular, patients who received paracetamol were more likely to be post-operative admissions, had higher maximum temperature values and were more likely to have fever. Among study patients, 4,397 (27.8%) had at least one episode of temperature >38.0°C during their ICU stay. An infection-related admission diagnosis was present in a similar percentage of patients in both groups.

**Table 1:** **Comparison of the characteristics of patients who received or did not receive at least one dose of paracetamol** ^**a**^

	**All patients, N =15,818**	**No paracetamol, n =5,772**	**Paracetamol, n =10,046**	***P*** **-value**
Age, yr	64 (51 to 73)	62 (49 to 73)	64 (52 to 73)	<0.001
Male sex	64% (1,019)	63% (3,631)	65% (6,568)	0.002
APACHE II score	16.9 (7.18)	17.7 (8.4)	16.5 (6.3)	<0.001
SAPS II score	29.5 (22.4)	28.3 (26.2)	30.2 (19.8)	<0.001
Hospital A	20.3% (3,206)	14% (786)	24% (2,419)	<0.001
Hospital B	10.9% (1,718)	11% (631)	11% (1,087)	0.83
Hospital C	10.2% (1,607)	12% (707)	9% (900)	<0.001
Hospital D	58.7% (9,288)	63% (3,648)	56% (5,640)	<0.001
Mechanical ventilation^b^	73% (11,513)	70% (4,032)	74% (7,481)	<0.001
Acute renal failure^c^	6% (944)	8% (477)	5% (457)	<0.001
Surgical admissions^d^	63% (9,994)	51% (2,949)	70% (7,045)	<0.001
Elective surgery	49% (7,707)	37% (2,154)	55% (5,553)	<0.001
Infection as admission diagnosis^e^	9% (1,419)	9% (518)	9% (901)	0.99
Number of temperature measurements	15 (9 to 36)	11 (5 to 20)	18 (11 to 49)	<0.001
Mean temperature, °C	36.7 (0.7)	36.6 (0.8)	36.8 (0.6)	<0.001
Highest temperature, °C	37.6 (1.0)	37.4 (0.9)	37.8 (1.0)	<0.001
Lowest temperature, °C	35.4 (2.2)	35.6 (1.9)	35.4 (2.4)	<0.001
SD of temperature, °C	0.62 (0.46)	0.59 (0.50)	0.64 (0.43)	<0.001
Temperature >38°C^f^	28% (4,397)	18% (984)	34% (3,413)	<0.001
Temperature >38.3°C^f^	20.3% (3,212)	11% (647)	26% (2,565)	<0.001
Temperature >38.5°C^f^	15.5% (2,456)	7% (429)	20% (2,027)	<0.001
Temperature >39°C^f^	7.9% (1,247)	3% (174)	11% (1,073)	<0.001
Temperature <35°C^f^	37.6% (5,949)	33% (1,927)	40% (4,022)	<0.001
Total dose, g	1 (0 to 4)	0 (0 to 0)	3 (2 to 7)	<0.001
Mean daily dose,^g^ g	1 (0 to 2)	0 (0 to 0)	1.9 (1.0 to 2.6)	<0.001

^a^APACHE, Acute Physiology and Chronic Health Evaluation; SAPS, Simplified Acute Physiology Score; SD, Standard deviation. ^b^Mechanical ventilation within first 24 hours of ICU admission. ^c^24-hour urine output is <410 ml AND creatinine >133 μmol/L AND patient is not receiving chronic dialysis. ^d^APACHE III-J principal postoperative diagnosis. ^e^A list of admission diagnosis with infection is provided in Additional file 1. ^f^Indicates the presence of a given temperature at any time during intensive care unit stay. ^g^Mean daily dose calculated by dividing total dose by number by treatment days.

### Outcomes

Overall, 2,164 patients (14%) died during hospitalization (Table 2). Survivors had a lower maximum temperature, were less likely to have a temperature >38.0°C and were also more likely to receive paracetamol (Table 2).

**Table 2:** **Baseline characteristics of the study patients according to survival** ^**a**^

	**Survivors, N =13,654**	**Dead, N =2,164**	***P*** **-value**
Age, yr	63 (50–73)	67 (55–76)	<0.001
Male sex	65% (8827)	63% (1372)	0.26
APACHE II score	15.46 (5.78)	26.15 (8.13)	<0.001
SAPS II score	26.9 (20.6)	45.5 (26.1))	<0.001
Hospital A	21% (2870)	15% (335)	<0.001
Hospital B	11% (1474)	11% (244)	0.5
Hospital C	10%(1300)	14% (307)	<0.001
Hospital D	59%(8010)	59% (1278)	0.73
Mechanical ventilation^b^	71% (9694)	84% (1819)	<0.001
Acute renal failure^c^	71% (9694)	84% (1819)	<0.001
Surgical admissions^d^	4% (572)	17% (362)	<0.001
Elective surgery	69% (9398)	28% (596)	<0.001
Infection as admission diagnosis^e^	55% (7507)	9% (200)	<0.001
Number of temperature measurements	8% (1027)	18% (392)	<0.001
Mean temperature, °C	36.7 (0.6)	36.6 (1.0)	<0.001
Highest temperature, °C	37.6 (0.9)	37.9 (1.4)	<0.001
Lowest temperature, °C	35.5 (2.1)	34.9 (2.6)	<0.001
SD of temperature, °C	0.60 (0.45)	0.78 (0.49)	<0.001
Temperature >38°C^f^	26% (3469)	45% (928)	<0.001
Temperature >38.3°C^f^	18% (2441)	36% (771)	<0.001
Temperature >38.5°C^f^	13% (1812)	30% (644)	<0.001
Temperature >39°C^f^	6% (859)	18% (388)	<0.001
Temperature <35°C^f^	35% (4823)	52% (1126)	<0.001
Number of patients given paracetamol	66% (9050)	46% (996)	<0.001
Total dose, g	0 (0–0)	3 (2–7)	<0.001
Mean daily dose,^g^ g	0 (0–0)	1.9 (1.0-2.6)	<0.001

^a^APACHE, Acute Physiology and Chronic Health Evaluation; SAPS, Simplified Acute Physiology Score; SD, Standard deviation; ^b^Mechanical ventilation within first 24 hours of ICU admission; ^c^24 hour urine output is <410 ml AND creatinine >133 μmol/L AND patient is not receiving chronic dialysis. ^d^APACHE III-J principal postoperative diagnosis. ^e^A list of admission diagnosis with infection is provided in Additional file 1. ^f^Indicates the presence of a given temperature at any time during intensive care unit stay. ^g^Mean daily dose calculated by dividing total dose by number by treatment days.

**Figure 1:**
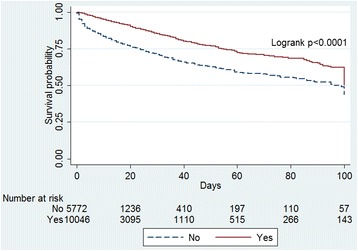
**Kaplan-Meier survival plot for all patients according to any exposure to paracetamol (yes) or no exposure to paracetamol (no).**

Administration of paracetamol was associated with an unadjusted reduction of in-hospital mortality (996 (9.9%) vs. 1,168 (20.1%) deaths; *P* <0.0001) and ICU mortality (530 (5.3%) vs. 858 (14.9%) deaths; *P* <0.0001). The Kaplan-Meier survival plot for all study patients is shown in Figure 1.

In multivariate logistic regression analysis, administration of paracetamol was significantly and independently associated with reduced in-hospital mortality (adjusted OR =0.60 (95% CI), 0.53 to 0.68), *P* <0.001) (Table 3). In addition, Cox proportional hazards regression modeling showed that patients who received paracetamol had a significantly longer time to death (adjusted HR =0.51 (95% CI, 0.46 to 0.56), *P* <0.001) (Table 3).

**Table 3:** **Adjusted odds ratios and hazards ratios for in-hospital mortality with the binary factor of paracetamol administration** ^**a**^

	**N**	**Adjusted OR (95% CI)**	***P*** **-value**	**Adjusted HR (95% CI)**	***P*** **-value**
All^b^	15,818	0.60 (0.53 to 0.68)	<0.001	0.51 (0.46 to 0.56)	<0.0001
Surgery^c^					
Yes	9,994	0.72 (0.58 to 0.91)	0.006	0.51 (0.42 to 0.61)	<0.0001
No	5,824	0.56 (0.48 to 0.66)	<0.001	0.51 (0.45 to 0.57)	<0.0001
Fever^d^					
Yes	4,397	0.76 (0.61 to 0.93)	0.009	0.57 (0.49 to 0.66)	<0.0001
No	11,421	0.54 (0.46 to 0.64)	<0.001	0.49 (0.43 to 0.56)	<0.0001
Medical, fever and infection^e^	681	0.67 (0.42 to 1.05)	0.08	0.47 (0.33 to 0.65)	<0.0001

^a^CI, Confidence interval; HR, Hazard ratio; OR, Odds ratio. ^b^Adjusted for Acute Physiology and Chronic Health Evaluation (APACHE) II score, hospital, surgical patient, infection as admission diagnosis, presence of fever, APACHE III admission diagnosis group, treatment limitation and propensity score (for receiving paracetamol). ^c^Adjusted for APACHE II score, hospital, infection as admission diagnosis, presence of fever, APACHE III admission diagnosis group, treatment limitation and propensity score (for receiving paracetamol). ^d^Adjusted for APACHE II score, hospital, surgical patient, infection as admission diagnosis, APACHE III admission diagnosis group, treatment limitation and propensity score (for receiving paracetamol). ^e^Adjusted for APACHE II score, hospital, APACHE III admission diagnosis group, treatment limitation and propensity score (for receiving paracetamol).

### Subgroup and sensitivity analysis

**Figure 2:**
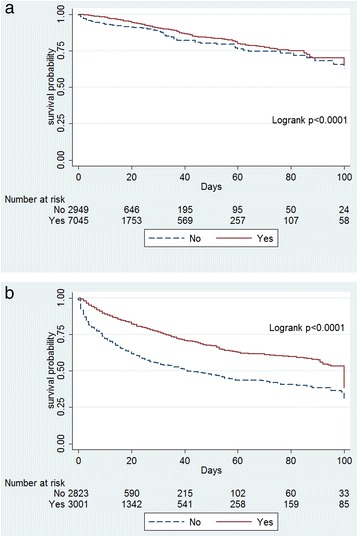
**Kaplan-Meier survival plots. (a)** Plot for surgical patients according to any exposure to paracetamol (yes) or no exposure to paracetamol (no). **(b)** Plot for medical patients according to any exposure to paracetamol (yes) or no exposure to paracetamol (no).

**Figure 3:**
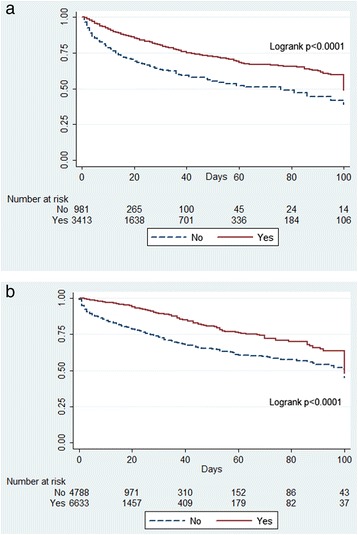
**Kaplan-Meier survival plots. (a)** Plot for patients with fever according to any exposure to paracetamol (yes) or no exposure to paracetamol (no). **(a)** Plot for patients without fever according to any exposure to paracetamol (yes) or no exposure to paracetamol (no).

**Figure 4:**
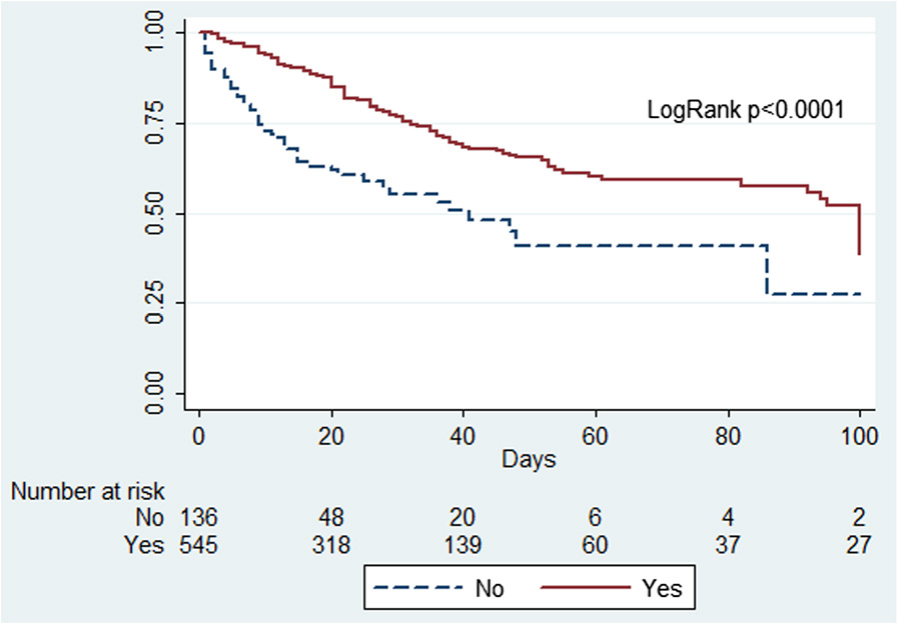
**Kaplan-Meier survival plot for medical patients with an infection-related admission diagnosis and a fever according to any exposure to paracetamol (yes) or no exposure to paracetamol (no).**

In surgical patients, administration of paracetamol remained a significant predictor of better outcome (Table 3, Figure 2a). Similar findings were observed in medical patients, with increased survival in patients treated with paracetamol (Table 3, Figure 2b). In addition, paracetamol was significantly associated with better outcomes in patients with and without fever (Table 3; Figure 3a, b) and in patients with infection as the admission diagnosis (Figure 4).

Importantly, however, in medical patients with fever and infection as the admission diagnosis, after adjustment (including adjustment for propensity; see Additional file 1: Tables E1 to E4 for details), paracetamol was not associated with significantly increased survival (Table 3), but it was associated with prolonged time to death.

When paracetamol was entered into the Cox proportional hazards model as a time-dependent variable, the independent association between paracetamol and longer time to death diminished but remained significant, except in the presence of fever or in patients with the combination of medical admission, fever and infection, where it failed to reach the predetermined significance level of *P* <0.01 (Table 4). Moreover, when the model was repeated after excluding early deaths and discharges (<20 hours) and with paracetamol at a time-dependent variable and fever also as a time-dependent variable, the findings did not materially change (Additional file 1: Table E5). We also adjusted the above prediction model for other potential confounders. These included cirrhosis (which might inhibit the use of paracetamol), the use of intravenous vs. enteral paracetamol, the presence of different temperature levels and tertiles of APACHE II scores. These additional analyses identified that the association between paracetamol and decreased mortality lost significance after adjustment for temperature >38.3°C, >38.5°C or >39°C and after adjustment for the lowest two tertiles of APACHE II scores (Additional file 1: Table E6).

**Table 4:** **Adjusted hazards ratio for in-hospital mortality with paracetamol administration as a time-dependent variable** ^**a**^

	**N**	**Adjusted HR (95% CI)**	***P*** **-value**
All^b^	15,818	0.68 (0.61 to 0.75)	<0.001
Surgery^c^			
Yes	9,994	0.64 (0.53 to 0.77)	<0.001
No	5,824	0.72 (0.64 to 0.81)	<0.001
Fever^d^			
Yes	4,397	0.84 (0.72 to 0.98)	0.02
No	11,421	0.58 (0.50 to 0.66)	<0.001
Medical, fever and infection^e^	681	0.70 (0.49 to 0.99)	0.04

^a^CI, Confidence interval; HR, Hazard ratio. ^b^Adjusted for Acute Physiology and Chronic Health Evaluation (APACHE) II score, hospital, surgical patient, infection as admission diagnosis, presence of fever, APACHE III admission diagnosis group, treatment limitation and propensity score (for receiving paracetamol). ^c^Adjusted for APACHE II score, hospital, infection as admission diagnosis, presence of fever, APACHE III admission diagnosis group, treatment limitation and propensity score (for receiving paracetamol). ^d^Adjusted for APACHE II score, hospital, surgical patient, infection as admission diagnosis, APACHE III admission diagnosis group, treatment limitation and propensity score (for receiving paracetamol). ^e^Adjusted for APACHE II score, hospital, APACHE III admission diagnosis group, treatment limitation and propensity score (for receiving paracetamol).

## Discussion

### Key findings

We conducted a multicenter, retrospective, observational study in a large cohort of patients admitted to four ICUs to examine the relationship between the use of paracetamol and patient outcome. We found a significant and independent association between the use of paracetamol and reduced in-hospital mortality. Additionally, the association between paracetamol and mortality was broadly consistent across subgroups. However, the strength of this association was attenuated, both in patients with fever in general and, particularly when paracetamol was treated as a time-dependent variable, in patients with a temperature >38.3°C, in medical patients with fever and an infection-related admission diagnosis and in patients in the lower tertiles of APACHE II score. In addition, the association may have been affected by indication bias, despite multiple other adjustments.

### Relationship with previous studies

Researchers in a few previous studies have described the use of antipyretic therapy to treat fever in ICU patients. In studies done in North America [15], Australia [17] and New Zealand [6], acetaminophen (paracetamol) was given to 58% to 70% of ICU patients. In our study, consistent with previous reports, 64% of patients admitted to the ICU received paracetamol. This observation confirms the fact that paracetamol is one of the most common drugs prescribed in the ICU. However, such practice may vary from country to country. For example, a binational, prospective, observational study done in Korea and Japan showed that only 148 (10%) of 1,425 ICU patients admitted to the ICUs received any paracetamol [12]. To our knowledge, no other studies have systematically assessed paracetamol use in critically ill patients.

We found that paracetamol administration was independently associated with reduced in-hospital mortality in critically ill patients. This observation is not consistent with two recently published meta-analyses whose authors found antipyretic therapy was neither beneficial nor harmful in critically ill adults without neurological injury [15,18]. Since the publication of those meta-analyses, investigators in a small randomized controlled trial have assessed the impact of acetaminophen-based antipyretic therapy strategies on the outcomes of critically ill patients [3]. Although a preliminary pilot study, 28-day all-cause mortality was not significantly different between the aggressive (2.6 g of paracetamol administered) and permissive (no paracetamol) fever control group. More recently, paracetamol was assessed in a small single-center study in patients with sepsis and detectable plasma free hemoglobin [19]. In that study of 40 patients, treatment with paracetamol was associated with more favorable biochemical outcomes: a decrease in F-isoprostanes and serum creatinine in the first 2 to 3 days of treatment. Thus, the effect of paracetamol administration for fever control on patient-centered outcome in critically ill patients remains unclear. A large (n =700) randomized placebo-controlled trial investigating the safety and efficacy of paracetamol in febrile ICU patients with known or suspected infection is currently underway [20,21].

### Study implications

Our findings that paracetamol was associated with decreased mortality in each subgroup, even after adjusting for confounding factors, are of importance and are not in keeping with our hypothesis. The use of paracetamol for not only antipyretic but also analgesic effect is believed to be common but has not been evaluated as an independent risk factor for outcome in the ICU overall and in specific ICU subgroups. Our findings that close to two-thirds of our patients received paracetamol confirm that, at least in the Australian and New Zealand context, paracetamol prescription in the ICU is common. Our additional findings that its administration is independently associated with improved survival and that such association remains after the use of propensity analysis, adjustments for key outcome-predictive baseline variables, and consideration of paracetamol administration as a time-dependent variable all suggest the need to more formally evaluate the role of paracetamol therapy in ICU patients. In addition, the variability of this association when adjusted for the degree of fever or illness severity or the presence of suspected infection further invite assessment in such specific groups.

### Strengths and limitations

This study has several strengths. It involved more than 15,000 patients from 4 ICUs. This makes it the largest study of the overall use of paracetamol in the ICU so far. Moreover, by analyzing close to 700,000 temperature measurements it allowed the most extensive assessment of its use in association with fever in critically ill patients. Finally, our study included a multifaceted statistical assessment of the possible independent relationship between the use of paracetamol and outcome using multiple models and adjusting for illness severity and propensity to receive paracetamol as well as the time-dependent nature of its administration and the time-dependent impact of fever, suspected infection and illness severity on its prescription.

However, like other studies of associations based on large databases, the present study is limited by the nature of the data available. Thus, we are unable to comment on interventions that might have affected body temperature, such as renal replacement therapy or external cooling for cardiac arrest. The study is also limited by the fact that no causal inferences can be drawn from the observations reported, especially because they may be markedly affected by indication bias. Additionally, we were unable to determine whether paracetamol was given as an antipyretic or as an analgesic or both. To attenuate the impact of this limitation, we performed subgroup analysis to identify patients more likely to receive paracetamol as either an antipyretic (patients who had fever or infection or both) or analgesic (patients after surgery) separately. We found that even after we excluded surgical patients, to whom paracetamol was most likely to have been given as an analgesic medication, the association between paracetamol and greater survival remained. As paracetamol is commonly given to surgical patients for analgesia, and as surgical patients have a better prognosis than medical patients, paracetamol may have simply acted as a marker of populations with an overall better prognosis. However, we found that when medical patients with fever and infection as the admission diagnosis (where use of the drug as an analgesic seems unlikely) were analyzed separately, the association between paracetamol therapy and increased survival time remained. Moreover, its association with increased survival in such patients and in patients with higher levels of fever or lower levels of illness severity, although losing significance, trended in a similar direction up to a temperature of 39°C. It is possible that paracetamol may have led to decreased use of narcotics and that such narcotic sparing effects may have been beneficial. Unfortunately, we do not have information on the use of narcotics in our patients. It is also possible that paracetamol may have led to decreased use of non-steroidal anti-inflammatory drugs (NSAIDs) and that such NSAID-sparing effects may have been beneficial. Although we do not have information on the use of NSAIDs in our patients, all units, by policy, use such drugs very infrequently.

The opportunity to receive paracetamol in the ICU varies with time and survival. Patients who are discharged early from the ICU have less opportunity to receive paracetamol and are also more likely to survive to hospital discharge. This effect potentially makes it less likely for paracetamol to be associated with better survival. However, patients who die early in the ICU also have a decreased probability of receiving paracetamol in the ICU, thus influencing the association between paracetamol and survival in a favorable direction. To compensate for such confounders, we adjusted outcome models according to the propensity to receive paracetamol and entered paracetamol exposure as a time-dependent variable in our statistical models. Such models continued to broadly show a beneficial association between paracetamol administration and hospital survival. We then applied such models after early discharges and early deaths were removed. The findings were unaltered. We note, however, that we were unable to develop a multilevel propensity analysis based on exposure because specific dosages of paracetamol were not available.

We defined fever as a temperature >38.0°C in accordance with previous studies [17,18]. Although the Society of Critical Care Medicine and the Infectious Diseases Society of America recommend that critically ill patients with a temperature of at least 38.3°C be considered febrile [22], evidence supporting such recommendations is lacking. Indeed the definition of fever in the literature varies widely, from at least 37.5°C to >39.0°C [22,23]. We are unable to comment on site and mode of temperature measurement. However, the measurement techniques would have applied to patients receiving or not receiving paracetamol in a similar way and seem unlikely to have introduced bias. Of interest, however, only when paracetamol was treated as a time-dependent variable and fever was defined as a temperature >39°C was the beneficial trend associating paracetamol with decreased mortality reversed. It is in these febrile patients with likely infection that paracetamol is currently being tested against placebo in a multicenter phase IIb trial (ACTRN12612000513819) [20,21].

## Conclusions

Paracetamol administration was common in the study ICUs and was independently associated with reduced in-hospital mortality after adjustment for several potential confounders. This association was broadly consistent among various subgroups, such as surgical or medical patients, but was attenuated in patients with fever and in medical patients with an infection-related admission diagnosis, in those with higher levels of fever and in patients in the two lowest tertiles of APACHE scores. Within the limitations of a large, multicenter, observational study, our findings suggest that paracetamol therapy may have an independent relationship with patient outcome. Given how commonly paracetamol is prescribed, and on the basis of the evidence obtained in this study, a program leading to randomized controlled trials designed to investigate the impact of paracetamol administration on patient outcomes in ICU appears both rational and desirable.

## Key messages

- Among critically ill patients, little is known about the association between paracetamol administration and patient outcomes.
- We conducted a large multicenter study and found an independent association between paracetamol administration and greater survival to hospital discharge.
- This association applied to surgical and medical patients and to febrile and non-febrile patients.
- This association remained present after Cox proportional hazards analysis, propensity score adjustment, treatment of paracetamol administration as a time-varying variable and sensitivity analysis.

## Additional file

Additional file 1:
**Electronic appendix to paracetamol study.**

## Abbreviations

APACHE: Acute Physiology and Chronic Health Evaluation
CI: Confidence interval
HR: Hazard ratio
ICU: Intensive care unit
NSAID: Non-steroidal anti-inflammatory drug
OR: Odds ratio
SAPS: Simplified Acute Physiology Score
SD: Standard deviation
