# Supplementary material for: Paracetamol therapy and outcome of critically ill patients: a multicenter retrospective observational study
Source: Crit Care. 2015 Apr 13;19(1):162. doi: 10.1186/s13054-015-0865-1 (PMC4411740; doi:10.1186/s13054-015-0865-1)
Supplement: Additional file 1: — Electronic appendix to paracetamol study. [file 13054_2015_865_MOESM1_ESM.pdf]

Additional file 1 (pdf)

Title: Electronic appendix to paracetamol study

eTEXT 1.

APACHE admission diagnosis with infection

APACHE admission diagnosis consistent with infection:

- **nonoperative:** pneumonia, parasitic pneumonia, bacterial pneumonia, viral pneumonia, gastrointestinal tract perforation, gastrointestinal tract obstruction, neurologic infection, cellulitis or soft tissue infection;
- **postoperative:** respiratory infection, gastrointestinal tract perforation or rupture, cholecystitis or cholangitis, fistula or abscess surgery, peritonitis, cellulitis or soft tissue infection.

Figure E 1: Frequency distribution for the administration of the first dose of paracetamol*

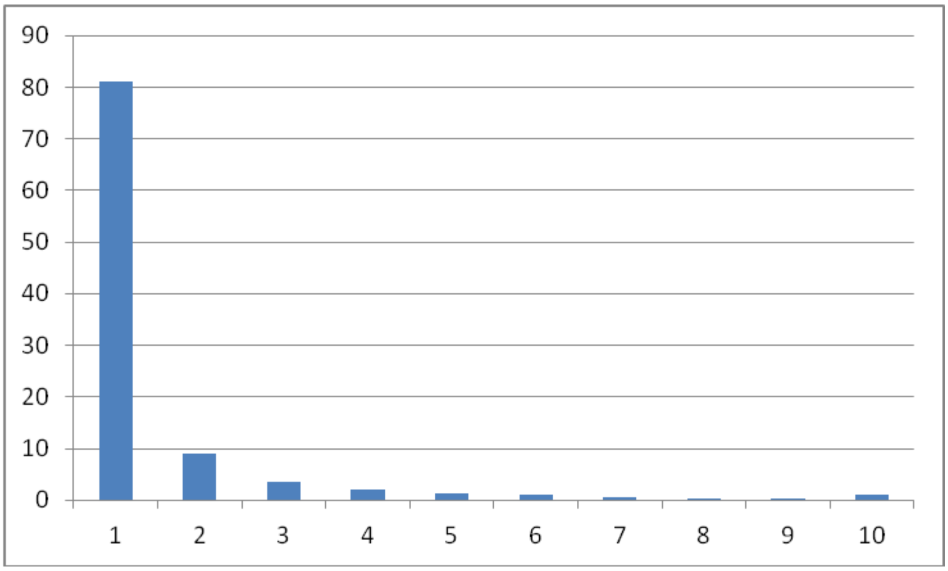

- 90% of patients treated with paracetamol had their first exposure in the first 2 days.

Table E-1: Variables retained in the propensity model to receive paracetamol during ICU stay

Variables	Odds Ratio (95% Confidence Interval)	P value
Admission date	1.001 (1.001-1.001)	<0.001
Age	1.003 (1.001-1.006)	0.009
Respiratory rate	1.007 (1.003-1.011)	<0.001
SAPS2	0.987 (0.985-0.989)	<0.001
Facility (Ref =Hospital D)		
Hospital A	0.852 (0.737-0.985)	0.030
Hospital B	0.581 (0.501-0.674)	<0.001
Hospital C	1.630 (1.422-1.869)	<0.001
Elective surgery	1.180 (1.050-1.327)	0.006
Intratracheal intubation	1.319 (1.022-1.704)	0.03
Acute renal failure[1]	0.820 (0.705-0.955)	0.01
Mechanical ventilation[2]	1.309 (1.014-1.691)	0.04
Number of comorbidities[3]	0.679 (0.616-0.748)	<0.001
Burns	0.364 (0.087-1.522)	0.16
Trauma	1.903 (1.532-2.365)	<0.001
Sepsis	1.704 (1.422-2.042)	<0.001
Cardiac arrest[4]	0.566 (0.458-0.701)	<0.001
Hospital admission Source: other hospital	1.227 (1.112-1.354)	<0.001
Source of admission to ICU		
Operating theatre	3.775 (2.768-5.148)	<0.001
Emergency department	1.693 (1.248-2.297)	0.001
Hospital floor	2.692 (1.973-3.674)	<0.001
Other hospital	2.335 (1.702-3.205)	<0.001
Treatment limitations on admission	0.382 (0.302-0.483)	<0.001
Primary diagnosis according to APACHE III classification[5]		
Cardiovascular (y/n)	1.385 (1.223-1.568)	<0.001
Gastrointestinal (y/n)	0.598 (0.522-0.684)	<0.001
Haematology (y/n)	1.882 (0.819-4.325)	0.13
Metabolic (y/n)	0.375 (0.305-0.461)	<0.001
Respiratory (y/n)	1.219 (1.061-1.401)	0.005

1. 24 hour urine output is < 410ml AND creatinine > 133 μmol/L AND patient is not receiving chronic dialysis.
2. Mechanical ventilation within 24 hours of ICU admission
3. AIDS, Hepatic failure, Lymphoma, Metastatic cancer, Leukaemia/Myeloma, Immunosuppressed, Cirrhosis

4. Cardiac arrest within the previous 24 hours
5. Covers both non-operative and post-operative admissions

Table E2 - Propensity model and matching

Existing propensity model

variable	OddsRatioEst	LowerCL	UpperCL	ProbChiSq
Age	1.00	1.00	1.01	0.0094
AP3_Cardiovasc	1.39	1.22	1.57	0.0000
AP3_Gastrointe	0.60	0.52	0.68	0.0000
AP3_Haematolog	1.88	0.82	4.32	0.1366
AP3_Metabolic	0.38	0.30	0.46	0.0000
AP3_Respirator	1.22	1.06	1.40	0.0052
AP3_sepsis	1.70	1.42	2.04	0.0000
AP3_Trauma	1.90	1.53	2.36	0.0000
ARF01	0.82	0.70	0.95	0.0107
Burns	0.36	0.09	1.52	0.1663
CardiacArrest	0.57	0.46	0.70	0.0000
COMORB7	0.68	0.62	0.75	0.0000
ELECT01	1.18	1.05	1.33	0.0055
HOS_ADM_OthHOS	1.23	1.11	1.35	0.0000
HOSPITAL_A	0.85	0.74	0.98	0.0302
HOSPITAL_B	0.58	0.50	0.67	0.0000
HOSPITAL_C	1.63	1.42	1.87	0.0000
ICU_AD_DT	1.00	1.00	1.00	0.0000
ICU_ADM_EMERG	1.69	1.25	2.30	0.0007
ICU_ADM_OT	3.77	2.77	5.15	0.0000
ICU_ADM_OthHOS	2.34	1.70	3.21	0.0000
ICU_ADM_WARD	2.69	1.97	3.67	0.0000
INTUBATED01	1.32	1.02	1.70	0.0336
RR	1.01	1.00	1.01	0.0003
SAPS2	0.99	0.98	0.99	0.0000
TreatLimitorPall	0.38	0.30	0.48	0.0000
VENTILATED01	1.31	1.01	1.69	0.0391

Table E3: Hazard ratios (95%CI) for Cox models (Panadol vs no Panadol) after excluding early deaths and discharges[1]

Categories	HR	low95%CI	High95%CI
All	0.646	0.581	0.717
Temp>38, medical admission & infection	0.699	0.49	0.997
Temp>38, no infection	0.874	0.729	1.047
by Surgery (No)	0.686	0.606	0.776
by Surgery (Yes)	0.602	0.49	0.738
by Temp>38 (No)	0.582	0.502	0.675
by Temp > 38 (Yes)	0.842	0.718	0.988
By diagnostic category			
Other	0.263	0.059	1.18
Trauma	0.501	0.253	0.992
Neurologic	0.586	0.46	0.745
Gastrointestinal	0.637	0.479	0.847
Cardiovascular	0.659	0.539	0.805
Sepsis	0.665	0.497	0.89
Respiratory	0.721	0.56	0.928
Renal	0.912	0.436	1.906
Metabolic	1.069	0.483	2.367

1. All patients with an ICU stay < 20 hours were excluded

Table E 4 – Propensity matching of cohorts

Comparison between matched cohorts

no paracetamol (n=4781)	paracetamol (n=4781)	
60.18 (16.57)	60.19 (18.7)	Age
38% (1798)	36% (1741)	AP3_Cardiovasc
18% (864)	18% (880)	AP3_Gastrointe
0% (10)	0% (8)	AP3_Haematolog
5% (253)	4% (187)	AP3_Metabolic
13% (620)	14% (673)	AP3_Respirator
6% (306)	7% (340)	AP3_sepsis
3% (161)	4% (174)	AP3_Trauma
8% (359)	7% (339)	ARF01
0% (2)	0% (4)	Burns
4% (206)	4% (194)	CardiacArrest
0 [0-0]	0 [0-0]	COMORB7
40% (1908)	40% (1910)	ELECT01
26% (1246)	27% (1295)	HOS_ADM_OthHOS
12% (575)	12% (582)	HOSPITAL_A
13% (610)	13% (621)	HOSPITAL_B
60% (2854)	55% (2606)	HOSPITAL_C
17262.45 (1214.32)	17392.41 (1038.74)	ICU_AD_DT
16% (779)	15% (738)	ICU_ADM_EMERG
53% (2518)	53% (2557)	ICU_ADM_OT
14% (649)	14% (653)	ICU_ADM_OthHOS
15% (736)	15% (739)	ICU_ADM_WARD
71% (3385)	71% (3379)	INTUBATED01
14 [10-26]	14 [10-26]	RR
27 [5-43]	29 [13-42]	SAPS2
3% (160)	3% (122)	TreatLimitorPall
70% (3350)	70% (3331)	VENTILATED01

Details of propensity matching

Overall, 4781/5772 (83%) of patients that did not receive paracetamol were matched on a 1:1 basis with 4781 patients that did receive paracetamol.
Patients were matched via propensity score with each patient matched to within +/-5%.

Details of matched analysis
4781/5772 (83%) of patients that did not receive paracetamol were matched on a 1:1 basis with 4781 patients that did receive paracetamol.
Patients were matched via propensity score with each patient matched to within +/-5%.

As can be seen in Table 1, patients were reasonably-well matched for the confounding variables that were used to develop the propensity score.
Matched analysis was performed using logistic regression and Cox-proportional hazards regression with adjustment for matching strata.

Results are present as raw and adjusted Odds Ratios (Hospital mortality) (adjusting for APACHE II, hospital, surgical patient, infection as admission diagnosis, presence of fever, APACHE III admission diagnosis group, treatment limitation

Results are also present as raw and adjusted Hazard Ratios (Time to death) (adjusting for APACHE II, hospital, surgical patient, infection as admission diagnosis, presence of fever, APACHE III admission diagnosis group, treatment limitation) with paracetamol usage treated as a time dependent variable.

**OR raw**	**P-raw**	**OR adj**	**P-adj**	**HR raw**	**P-raw**	**HR adj**	**p adj**
0.67 (0.60-0.75)	<0.0001	0.57 (0.46-0.71)	<0.0001	0.51 (0.45-0.59)	<0.0001	0.68 (0.54-0.87)	0.002

Table E5: Adjusted odds ratio and hazards ratio for in-hospital mortality with paracetamol administration as a time-dependent variable

	N	Adjusted HR (95%CI)	P value
All [a]	15818	0.68 (0.61-0.75)	<0.0001
Surgery [b]			
Yes	9994	0.64 (0.53-0.77)	<0.0001
No	5824	0.72 (0.64-0.81)	<0.0001
Fever [c]			
Yes	4397	0.84 (0.72-0.98)	<0.0239
No	11421	0.58 (0.50-0.66)	<0.0001
Medical, fever_and_infection [d]	681	0.70 (0.49-0.99)	<0.0416

Table E6

Multivariable Cox proportional hazards model with paracetamol use as a time dependent parameter with adjustments for form of delivery (intravenous [IV] or enteral), different body temperature at any time, different APACHE score tertiles and presence or absence of cirrhosis. These data are to be seen as additional to the previous model upon which they are superimposed.

Parameter	Estimate	SE	Chi sq.	Prob	HR	HRLCL	HRUCL	global P	Model
panUsage_TimeDepend	-0.39	0.05	59.26	0.00	**0.68**	**0.61**	**0.75**	**0.000**	**All**
panUsage_TimeDepend	-0.51	0.10	28.92	0.00	**0.60**	**0.50**	**0.72**	**0.000**	**IV delivery only**
panUsage_TimeDepend	-0.37	0.05	48.43	0.00	**0.69**	**0.62**	**0.76**	**0.000**	**enteral delivery only**
panUsage_TimeDepend	-0.43	0.07	34.30	0.00	**0.65**	**0.57**	**0.75**	**0.000**	**TempUnd35_any**
panUsage_TimeDepend	-0.36	0.18	4.15	0.04	**0.70**	**0.49**	**0.99**	**0.042**	**Temp>38, medical & infect**
panUsage_TimeDepend	-0.32	0.06	28.95	0.00	**0.72**	**0.64**	**0.81**	**0.000**	**by Surgery (no)**
panUsage_TimeDepend	-0.45	0.10	21.40	0.00	**0.64**	**0.53**	**0.77**	**0.000**	**by Surgery (yes)**
panUsage_TimeDepend	-0.54	0.06	70.91	0.00	**0.58**	**0.52**	**0.66**	**0.000**	**by TempOv38.3_Any (no)**
panUsage_TimeDepend	-0.12	0.09	1.84	0.18	**0.88**	**0.74**	**1.06**	**0.175**	**by TempOv38.3_Any (yes)**
panUsage_TimeDepend	-0.52	0.06	74.96	0.00	**0.59**	**0.53**	**0.67**	**0.000**	**by TempOv38.5_Any (no)**
panUsage_TimeDepend	-0.07	0.10	0.41	0.52	**0.94**	**0.77**	**1.15**	**0.522**	**by TempOv38.5_Any (yes)**
panUsage_TimeDepend	-0.55	0.07	60.25	0.00	**0.58**	**0.50**	**0.66**	**0.000**	**by TempOv38_Any (no)**
panUsage_TimeDepend	-0.18	0.08	5.10	0.02	**0.84**	**0.72**	**0.98**	**0.024**	**by TempOv38_Any (yes)**
panUsage_TimeDepend	-0.48	0.05	77.59	0.00	**0.62**	**0.56**	**0.69**	**0.000**	**by TempOv39_Any (no)**
panUsage_TimeDepend	0.11	0.15	0.58	0.45	**1.12**	**0.84**	**1.49**	**0.446**	**by TempOv39_Any (yes)**
panUsage_TimeDepend	-0.33	0.23	2.02	0.16	**0.72**	**0.46**	**1.13**	**0.155**	**by apachetert (ap2=0-13)**
panUsage_TimeDepend	-0.21	0.15	1.96	0.16	**0.81**	**0.61**	**1.09**	**0.162**	**by apachetert (ap2=13-18)**
panUsage_TimeDepend	-0.42	0.06	55.73	0.00	**0.66**	**0.59**	**0.73**	**0.000**	**by apachetert (ap2>18)**
panUsage_TimeDepend	-0.43	0.05	66.56	0.00	**0.65**	**0.59**	**0.72**	**0.000**	**by cirrhosis (no)**
panUsage_TimeDepend	-0.77	0.19	17.29	0.00	**0.46**	**0.32**	**0.66**	**0.000**	**by cirrhosis (yes)**

Temp Und35_any = any episode of temperature under 35°C
Temp Ov38.3_Any = any episode of temperature over 38.3°C
Temp Ov38.5_Any = any episode of temperature over 38.5°C
Temp Ov39_Any = any episode of temperature over 39°C
Apachetert = APACHE II value tertile
Ap2 = APACHE II score
